# A Whole-Brain Topographic Ontology

**DOI:** 10.1146/annurev-neuro-082823-073701

**Published:** 2024-07-01

**Authors:** Michael Arcaro, Margaret Livingstone

**Affiliations:** 1 Department of Psychology, University of Pennsylvania, Philadelphia, Pennsylvania, USA; 2 Department of Neurobiology, Harvard Medical School, Boston, Massachusetts, USA

**Keywords:** topography, development, domain specificity, experience

## Abstract

It is a common view that the intricate array of specialized domains in the ventral visual pathway is innately prespecified. What this review postulates is that it is not. We explore the origins of domain specificity, hypothesizing that the adult brain emerges from an interplay between a domain-general map-based architecture, shaped by intrinsic mechanisms, and experience. We argue that the most fundamental innate organization of cortex in general, and not just the visual pathway, is a map-based topography that governs how the environment maps onto the brain, how brain areas interconnect, and ultimately, how the brain processes information.

## 1. INTRODUCTION

Decades of research have established the pervasive notion that the primate ventral cortex comprises discrete specialized areas, each dedicated to processing different biologically important categories such as faces, body parts, objects, and places. The development of these category-selective domains has been a focus of intense debate over whether these functions are present at birth, programmed to develop as a consequence of evolutionary adaptation, or reflect a general-purpose architecture that adapts to its specific environment within an individual’s lifetime. For example, faces are believed to have a special neural status because of their role in social behavior. Face domains are absent in neonate macaques and require face-specific experience to develop. However, primate neonates show a looking preference for face-like images. Additionally, cross-modal category specificity, including for faces, is present in the temporal lobe of congenitally blind individuals. These seemingly contradictory findings underscore the complexity of the nature-nurture interplay. Here, we review the development of category-selective domains. We argue that these regions are not circuits specialized by evolutionary adaptation but emerge from a combination of innate, general-purpose architecture and environment-driven self-organization. We further propose that this framework applies to specializations throughout the entire cortex. Our goal is to integrate findings across the field pertaining not only to the development of category-selective domains in the ventral visual cortex but also to the fundamental principles guiding cortical specialization in general.

## 2. CATEGORICAL DOMAINS

**Figure 1:**
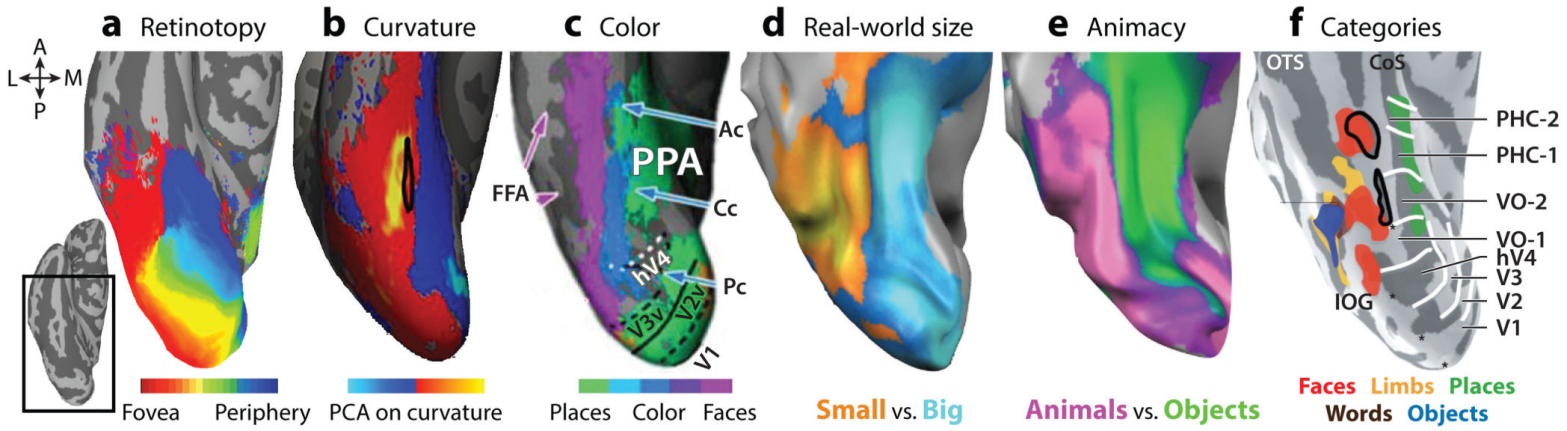
Topographic organization of VTC. In cortical surface reconstructions of the human VTC, several organizing frameworks have been observed, including (*a*) retinotopy (Wang et al. 2015), (*b*) shape/curvature, (*c*) color, (*d*) object size, (*e*) animacy, and ( *f* ) distinct category-selective domains. Abbreviations: A, anterior; Ac, anterior color; Cc, central color; CoS, collateral sulcus; FFA, fusiform face area; hV4, human V4; IOG, inferior occipital gyrus; L, lateral; M, medial; OTS, occipitotemporal sulcus; P, posterior; Pc, posterior color; PCA, principal components analysis; PHC, parahippocampal cortex; PPA, parahippocampal place area; V, ventral; VO, ventral occipital; VTC, ventral temporal cortex. Panel *b* adapted with permission from Yue et al. (2020), panel *c* adapted from Lafer-Sousa et al. (2016) (CC BY 4.0), panels *d* and *e* adapted from Konkle & Caramazza (2013) (CC BY-NC-SA 3.0), and panel *f* adapted with permission from Grill-Spector & Weiner (2014).

The primate ventral visual cortex is vital for visual object recognition. Neurological studies and functional magnetic resonance imaging (fMRI) have established that inferior temporal (IT) cortex in macaque monkeys and ventral temporal cortex (VTC) in humans have subdivisions, or domains, specialized in processing specific categories such as faces, places, text, bodies, or tools. The consistent anatomical positioning of these domains in adults and their presence across primate species indicate strong developmental constraints from genetics or regularities of experience. However, the ontology of this intrinsic organization is difficult to interpret from studying adult brains. Several organizing principles for the primate ventral visual cortex have been proposed based on observations in adults (Figure 1). These principles include categories (Downing et al. 2006), animacy (Konkle & Caramazza 2013), retinotopy (Hasson et al. 2002), real-world size (Konkle & Oliva 2012), texture (Long et al. 2018), shape features (Bao et al. 2020, Op de Beeck et al. 2008, Yue et al. 2020), and color (Lafer-Sousa et al. 2016). All these features correlate with each other. For example, face domains encode warm-colored animate images and are found in cortical regions that represent central visual space and are responsive to curvy shapes (Hasson et al. 2002, Lafer-Sousa & Conway 2013, Srihasam et al. 2014, Yue et al. 2020). In contrast, scene domains encode inanimate categories, are found in cortex representing the periphery, and prefer rectilinear shapes (Nasr et al. 2014, Srihasam et al. 2014). These interrelationships between feature dimensions in adults make it difficult to resolve which ones primarily drive the development of ventral visual cortex organization.

Adding to the complexity of category domain organization, the anatomical localization of category domains differs across primates. In humans, these regions are primarily within and around fusiform cortex, whereas in macaques they are found within and around the superior temporal sulcus (STS). Thus, the developmental mechanisms guiding domain formation must be flexible enough to account for such species-specific differences. Moreover, the presence of a text domain that emerges later in human postnatal development, and only after extensive experience, implies that environmental factors play a pivotal role in the formation of at least some domains. Given that primates have substantial early exposure with many categories ( Jayaraman et al. 2017), environmental influences could determine the development of other category specializations. These observations highlight the necessity of uncovering the developmental trajectory of these specialized circuits.

## 3. DEVELOPMENT OF CATEGORY DOMAINS

### 3.1. Face Domain Development

While face-selective neurons have been detected in 5.5-week-old macaques (Rodman et al. 1993), face-selective patches—comprising hundreds of thousands of neurons—are not observed with fMRI for the first several months (Livingstone et al. 2017). This apparent discrepancy might be due to metabolic immaturity (Distler et al. 1996) or differences in tuning specificity. Neurons responsive to face-like shapes could reflect a feedforward low-level input convergence (Baek et al. 2021). Positron-emission tomography imaging studies of 2-month-old human infants showed early preferential responses to faces as compared to flashing lights in a region of VTC that is face-selective in adults (Tzourio-Mazoyer et al. 2002). However, the comparison stimuli were insufficient to test for face-specific responsiveness, and face responses appeared in cortical regions that lack face selectivity in adults. Human fMRI studies in 3-to-6-month-old infants found face-movie responses greater than scene-movie responses in fusiform cortex, lateral occipital cortex, and STS regions that are typically selective for faces in adults (Deen et al. 2017, Kosakowski et al. 2022). Similarly, our infant macaque fMRI study (Livingstone et al. 2017) identified early selectivity for face movies compared to scene movies but no selectivity for faces compared to objects until later in development. Rather than reflecting face-specific activity, the early differential responses to face movies in both studies may have been driven by image statistics and retinotopically specific motion energy. The earliest selectivity using static face images, which are typically used to identify face domains in adults, was approximately 200 days in macaques. Despite differences in interpretation, these results converge on the bigger picture that selectivity for visual features that differ across categories emerges within the initial postnatal months.

Although face domains emerge within the first postnatal year, their selectivity develops through adolescence. By the age of six, the large-scale spatial organization of face- and other category-selective regions is present in the human VTC (Scherf et al. 2007). The size of face-selective regions continues to increase into adulthood, correlating with improvements in recognition memory of faces (Golarai et al. 2007). Perceptual expertise, such as differentiating between own-race and other-race faces, continues to develop into adulthood (Golarai et al. 2021). Hence, postnatal development of face processing extends well past the initial emergence of face selectivity.

### 3.2. Effect of Visual Experience of Faces

The lack of face domains at birth in monkeys indicates that early visual experience could play a role in their formation. Supporting the potential role of experience, monkeys preferentially look at faces of species they visually experience early in postnatal development, even more than conspecific faces (Sugita 2008). Also, early human visual experience is enriched in faces (Jayaraman et al. 2017), and the extent of exposure predicts a neonate’s preference for their mother’s face over another’s (Bushnell 2001).

To resolve whether experience is necessary for face-domain formation, we reared macaque monkeys from birth, caring for them while wearing masks to hide our faces, but not our bodies and hands (Arcaro et al. 2017). Room dividers prevented them from seeing other monkeys in the colony room while still exposing them to sounds and scents of other monkeys. After the yearlong controlled rearing, fMRI showed that these monkeys lacked face domains in IT. However, domains for experienced categories, such as hands, bodies, and scenes, developed in the same parts of IT where these regions form in normally reared monkeys. Thus, the lack of face visual experience specifically impacted the development of face-selective regions without impairing other visual category–selective regions. Even when later exposed to faces, face-selective regions never became as strong as in normally reared animals, indicating either a critical developmental window or a significant difference in their visual diet. In contrast to control monkeys, which preferentially looked at faces when free viewing, face-deprived monkeys looked preferentially at hands. These findings provide causal evidence that postnatal visual experience is necessary for the development of face domains. Computational modeling further supports the idea that visual experience is sufficient for the emergence of specialized face domains (Blauch et al. 2022).

### 3.3. Role of Experience in Domain Formation Beyond Faces

The necessity of visual experience for the development of face domains likely exemplifies a general mechanism underlying the formation of specialized visual circuits. We found that young monkeys, when taught to discriminate human symbols, developed domains in stereotyped locations specifically responsive to the trained symbols (Srihasam et al. 2012), indicating that experience is sufficient for the formation of domains. This finding aligns with other observations: Literate humans, unlike illiterate individuals, possess specialized stereotyped regions for processing text (Cohen et al. 2000). And children who extensively played the video game Pokémon developed VTC regions selective for the game’s characters (Gomez et al. 2019). These findings support the idea that early visual experience and activity-dependent mechanisms drive the development of specialized circuits across primate ventral visual cortex. We attribute the stereotyped anatomical locations of these domains to an intrinsic architecture that constrains where specializations develop without being sufficient in itself for their development.

### 3.4. An Intrinsic Domain-General Topographic Architecture

**Figure 2:**
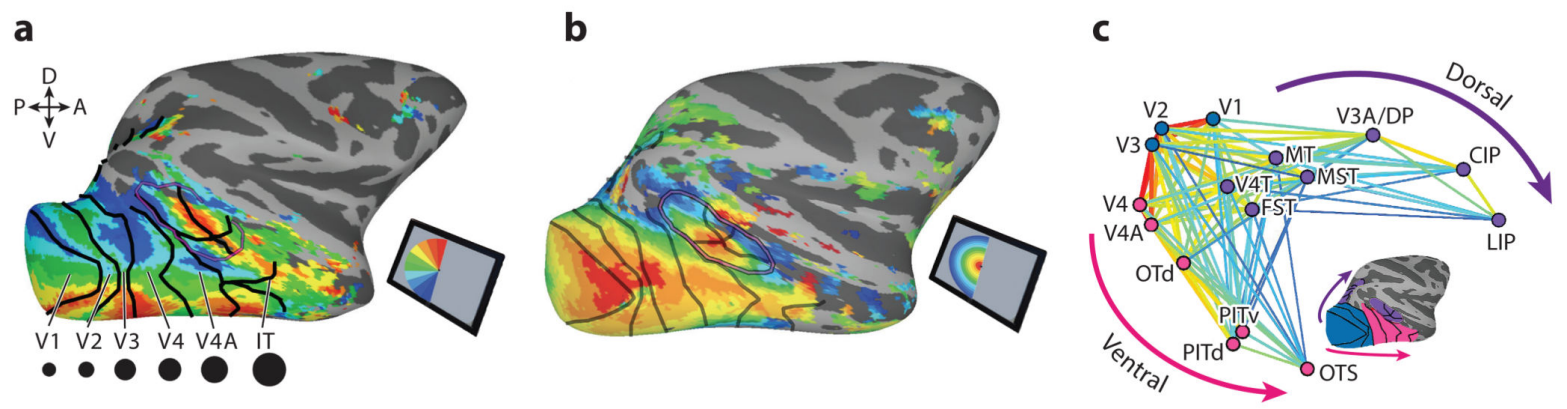
Maps, scale, and hierarchy. The primate visual system contains a hierarchically organized series of retinotopic maps that are also organized by selectivity for scale and shape. (*a*) Map of responsiveness to polar angle stimuli in the opposite visual field. (*b*) Map of responsiveness to stimulus eccentricity in the opposite visual field. (*c*) Hierarchical organization of the neonatal visual system, inferred from pairwise correlations between seed areas in functional MRI (Arcaro & Livingstone 2017a). Abbreviations: A, anterior; CIP, caudal intraparietal; D, dorsal; DP, dorsal prelunate; FST, fundus of the superior temporal sulcus; IT, inferior temporal; LIP, lateral intraparietal; MST, medial superior temporal; MT, middle temporal; OTd, occipitotemporal dorsal; OTS, occipitotemporal sulcus; P, posterior; PIT, posterior inferior temporal; V, ventral. Figure adapted from Arcaro & Livingstone (2017a).

Controlled rearing studies show that experience is both necessary and sufficient for category-selective domain formation but cannot account for the consistent anatomical positioning within a species. Any developmental theory must account for the stereotyped locations within a species and the varied anatomical localizations across species. Stereotyped locations for intensively experienced but entirely unnatural stimuli indicate that neuronal activity organizes inputs based on some kind of proto-organization. We argue that the primary components of this proto-organization are (*a*) topographic maps, orderly representations of sensory space along the cortical surface (Figure 2a,b), and (*b*) hierarchy, a series of integrative connections across these retinotopic maps (Figure 2c).

#### 3.4.1. Topographic maps of visual space.

Topographic maps of visual space are found throughout the primate visual system. Primary visual cortex (V1) receives input from the eyes via the thalamus and projects to adjacent cortex in a mirror-image fashion that links neurons representing overlapping parts of visual space (Tigges et al. 1981) and preserves the local continuity of the sensory space. This process of mirror mapping iterates throughout visual cortex (Figure 2a,b). Even higher visual areas once considered to lack spatial coding—such as human VTC and macaque IT—have extensive retinotopic maps (Arcaro & Livingstone 2017b, Janssens et al. 2014). Within each map, neurons often share distinct response properties (Zeki 1993), so topography reflects not only visual-field location but also functional specializations.

Topographic maps exist even before birth in V1 and are established by trophic factors (O’Leary et al. 1999) and refined through spontaneous activity (Cang et al. 2005b). Beyond V1, an extensive retinotopic organization is present in macaque (Arcaro & Livingstone 2017a) and human (Arcaro 2023, Kamps et al. 2020) neonates that is similar in structure to the adult organization. This robust retinotopic organization, apparent from the very earliest postnatal stages, precedes the emergence of face and other category domains. The persistence of retinotopic organization in congenitally blind humans (Butt et al. 2013, Striem-Amit et al. 2015) and in monkeys deprived of form vision (Arcaro et al. 2018) further attests to the intrinsic nature of retinotopic maps and the notion that the systematic representation of visual space is the primary organizing principle of the developing visual cortex. Thus, retinotopic mapping is a prominent feature of all visual cortex in the adult and is present from birth.

The retinotopic organization of primate ventral visual cortex can account for species-specific variations in the anatomical localization of category domains. In macaques, retinotopic maps extend from the occipital cortex into the STS, where face and body domains are typically found (Arcaro & Livingstone 2017b, Janssens et al. 2014, Kolster et al. 2014). In humans, maps extend from occipital cortex to category-selective regions in the fusiform gyrus and surrounding cortex (Arcaro et al. 2009, Kolster et al. 2010). Evolutionary adaptations influencing the location of sensory maps, such as the medial shift of the human V1 map (Arcaro et al. 2022), could explain interspecies differences in retinotopic, and thereby domain, localization.

#### 3.4.2. Ventral visual hierarchy.

Interareal connectivity organizes retinotopic maps into hierarchies. Within the ventral visual hierarchy, individual neurons in each map receive input from groups of neurons in the preceding map with overlapping visual-field representations. The tuning properties and spatial coverage of these inputs vary, resulting in increasingly larger and more complex representations of sensory space going up the hierarchy. Connections between maps are established prenatally (Baldwin et al. 2012, Batardiere et al. 2002). Moreover, the visual hierarchical organization is present in both macaque (Arcaro & Livingstone 2017a) and human (Arcaro 2023) neonates.

We propose that this intrinsic topographic hierarchy drives the stereotypical location of different category domains (Arcaro et al. 2019b). Imaging studies in macaques showed a robust correlation between retinotopy, spatial frequency, and curvature tuning across the visual system (Srihasam et al. 2014). Moreover, the correlation between curvature and spatial frequency is present in infant macaques and precedes the formation of domains (Arcaro & Livingstone 2017a). While the relationship between perceptual acuity, spatial frequency tuning, and eccentricity has been known since Euclid’s time, the correlation with curvature was a novel discovery. Correlations between eccentricity, curvature, and face selectivity (Yue et al. 2020) and between rectilinearity and scene selectivity (Nasr et al. 2014) also exist in adult humans. These findings suggest that a curvature bias in central visual field and a rectilinear bias in the periphery are intrinsic features of the primate ventral visual hierarchy.

The strong correlation between curvature and eccentricity indicates a prevalent end-stopping mechanism. End-stopped neurons respond maximally to short lines, with decreasing responsiveness for longer contours that extend into the inhibitory ends (Hubel & Wiesel 1965). These neurons are thought to support the identification of object boundaries and corners, which are information-rich image parts (Attneave 1954) and are therefore building blocks of shape and object vision. Because receptive field size and optimum length scale with eccentricity, central visual field neurons should be more responsive to high-curvature features, which are prevalent in objects like faces. Indeed, end-stopped neurons prefer curved over straight contours throughout the macaque ventral stream (Hubel & Livingstone 1987, Ponce et al. 2017). This makes sense considering that the length and width of receptive fields, and thus tuning for spatial frequency and stimulus length, both scale with eccentricity. Thus, an eccentricity map inherently imposes a bias for small (high spatial frequency), short (curvy) stimuli centrally and large (low spatial frequency), long (straight) stimuli peripherally. Thus, end-stopping provides a plausible mechanism underlying selectivity for high-curvature features over straight contours (Hubel & Livingstone 1987) in central visual field parts of IT, where face-selective domains develop. Thus, a retinotopic hierarchy enables the emergence of complex shape tuning in downstream areas without necessitating shape prespecification.

### 3.5. The Interplay Between Intrinsic and Experience-Driven Factors

Domain formation for complex visual features like faces is unlikely to be reducible to a single mechanism, since each stage in the visual hierarchy involves combinatorial computations. Category selectivity in IT aligns along dimensions of shape selectivity in monkeys (Bao et al. 2020) and humans (Coggan & Tong 2023). Yet, adult category selectivity, particularly in anterior areas, cannot be entirely explained by low- and mid-level shape features. We propose that postnatal activity-dependent plasticity refines intrinsic selectivity based on experience. Initial tuning for simple visual features can be modified through experience into an organization for behaviorally meaningful content by shifting or narrowing tuning. For instance, neurons in areas where face domains typically develop initially respond to round or concentric features found in both faces and objects like clocks. With experience, these neurons might come to respond more to faces and less to infrequently encountered or behaviorally unimportant objects, even if they share similar low-level features. Notably, in adult monkeys, face-selective neurons still respond to round objects. The presence of neonatal topographic maps selective for image scale and curvature underscores our hypothesis: Early shape biases in IT set the stage for specialized domain development. The refinement of tuning to heavily experienced features (such as faces) would not disrupt the underlying topographic shape map but could result in strengthening of selectivity for some features or categories compared to others.

**Figure 3:**
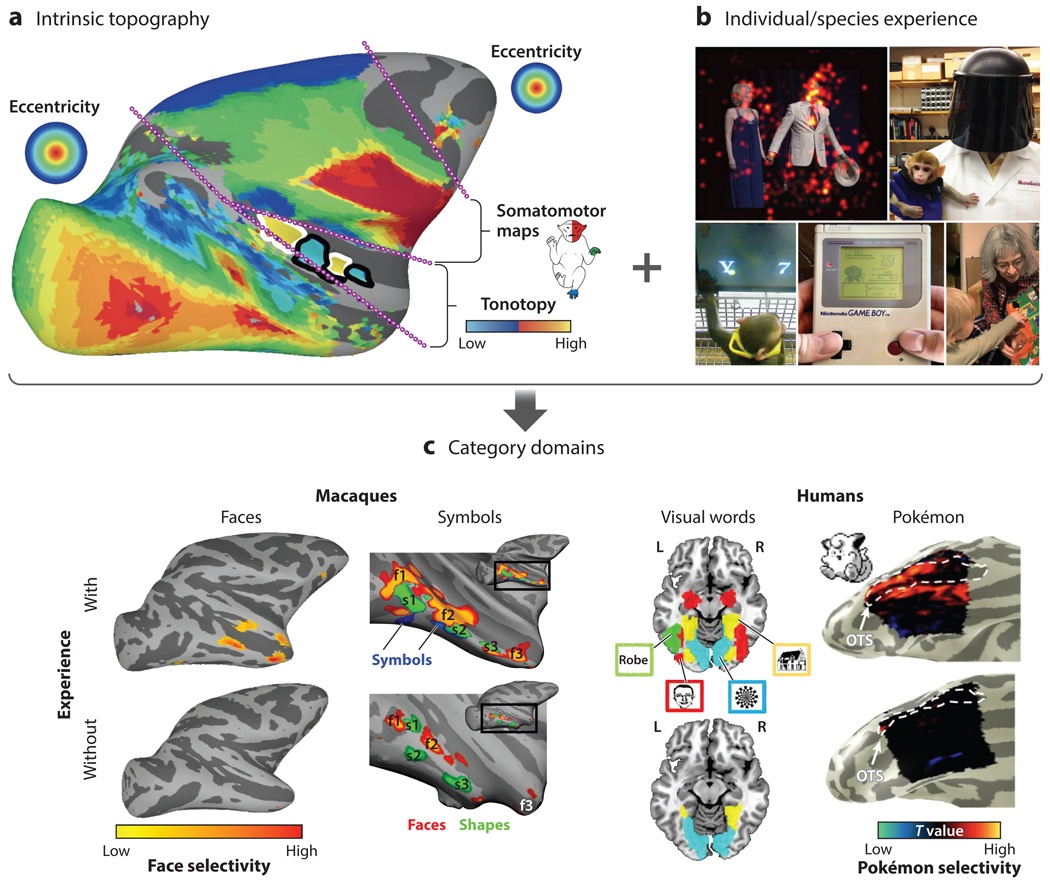
Activity-acting-on-maps hypothesis. (*a*) Topographic maps established before birth provide the scaffolding for individual- and species-specific experience-driven domain development. Colors in the visual pathway (to the *left* of the *first dotted line*) indicate eccentricity. Colors between the next set of dotted lines indicate tuning for auditory tonotopy. Colors between the next pair of dotted lines indicate somatomotor maps, as indicated on the monkey icon. Colors in the most anterior part of the map (the frontal eye fields) again indicate visual field eccentricity. Panel *a* adapted from Arcaro & Livingstone (2021). (*b*) Early visual experience acts on this intrinsic architecture to sculpt selectivity. (*Clockwise from upper left*) Early face looking behavior sculpts face domains (Arcaro et al. 2017) (photograph by A. Stubbs, provided by the Neural Correlate Society); face deprivation prevents the formation of face domains (Arcaro et al. 2017) (photograph provided by M. Livingstone); learning to read leads to the formation of text domains (Dehaene et al. 2010) (photograph by D. Livingstone); playing Pokémon produces Pokémon domains (Gomez et al. 2019) (photograph provided by J. Gomez); and symbol learning forms symbol domains in macaques (Srihasam et al. 2012) (photograph by M. Livingstone). (*c*) Various functionally specific domains in macaques (*left*) and humans (*right*) form as a consequence of experience acting on visual maps. (*Left* to *right*) Face domains are absent in monkeys who lack face experience (maps adapted from Arcaro & Livingstone 2021); symbol domains are present in monkeys who learn to distinguish human symbols (maps adapted from Srihasam et al. 2012); word and letter domains are present in humans who have learned to read (maps adapted with permission from Feng et al. 2022); Pokémon domains are found in humans who played Pokémon extensively as children (maps adapted with permission from Gomez et al. 2019).

Controlled rearing experiments demonstrate a key role for experience in domain development, yet visual input alone does not fully account for the anatomical consistency of domains. We hypothesize that visual experience, constrained by intrinsic topographies, generates functional specializations in fixed IT locations that align with species-specific architecture (Figure 3). An activity-acting-on-maps framework serves as a general model for cortical development, where an initial topographic architecture interacts with experience to generate specialized circuits. Next, we argue that the same principles apply to other domains and brain regions.

## 4. TOPOGRAPHIC PRINCIPLES OF DEVELOPMENT

### 4.1. Maps–Spam–Maps

Central to our hypothesis are the ideas that the brain is inherently structured to represent information topographically and that this architecture is fundamental for processing the sensory environment (Kaas 1997, Mountcastle 1957). A large expanse of cortex—spanning auditory, motor, somatosensory, and visual cortex—consists of orderly representations of our environment along sensory surfaces.

#### 4.1.1. Topographic maps as a ubiquitous principle of sensory systems.

Topographic maps are present at birth. In newborn macaques, the entire visual system consists of topographic maps (Arcaro & Livingstone 2017a). Both somatosensory and motor areas are also already organized at birth into multiple topographic body maps (Arcaro et al. 2019a, Dall’Orso et al. 2018). In between the visual field maps and body maps are multiple auditory maps of frequency, which we speculate are also present at birth. At birth, neocortex mirrors the spatial receptors of the sensory and motor periphery (Figure 3a). Thus, the developing cortex is already topographically organized to accurately sample from, respond to, and learn from the environment in a spatially appropriate way.

While postnatal experience can modify internal features of primary sensory maps, such as monocular deprivation restructuring V1’s ocular dominance organization, V1’s retinotopic organization remains consistent. Postnatal experience can modify the topology of human higher-order maps in the VTC, making text regions more foveal (Gomez et al. 2018); yet, the fundamental topography persists. Further, any alterations to the map of sensory space are minor compared to experience-driven changes in tuning. Even when sensory inputs are rerouted, such as directing retinal inputs to auditory thalamic structures, auditory cortex processes visual input conforming to its inherent organization (Roe et al. 1990). With peripheral sensory loss (Chino et al. 2001, Pons et al. 1991), representations of the surrounding cortex shift into the deprived cortex, still adhering to the overall topographic map. Only disruptions to genetic factors controlling cortical patterning distort the brain’s topographic structure (Bishop et al. 2002, Cang et al. 2005a).

#### 4.1.2. Map formation.

**Figure 4:**
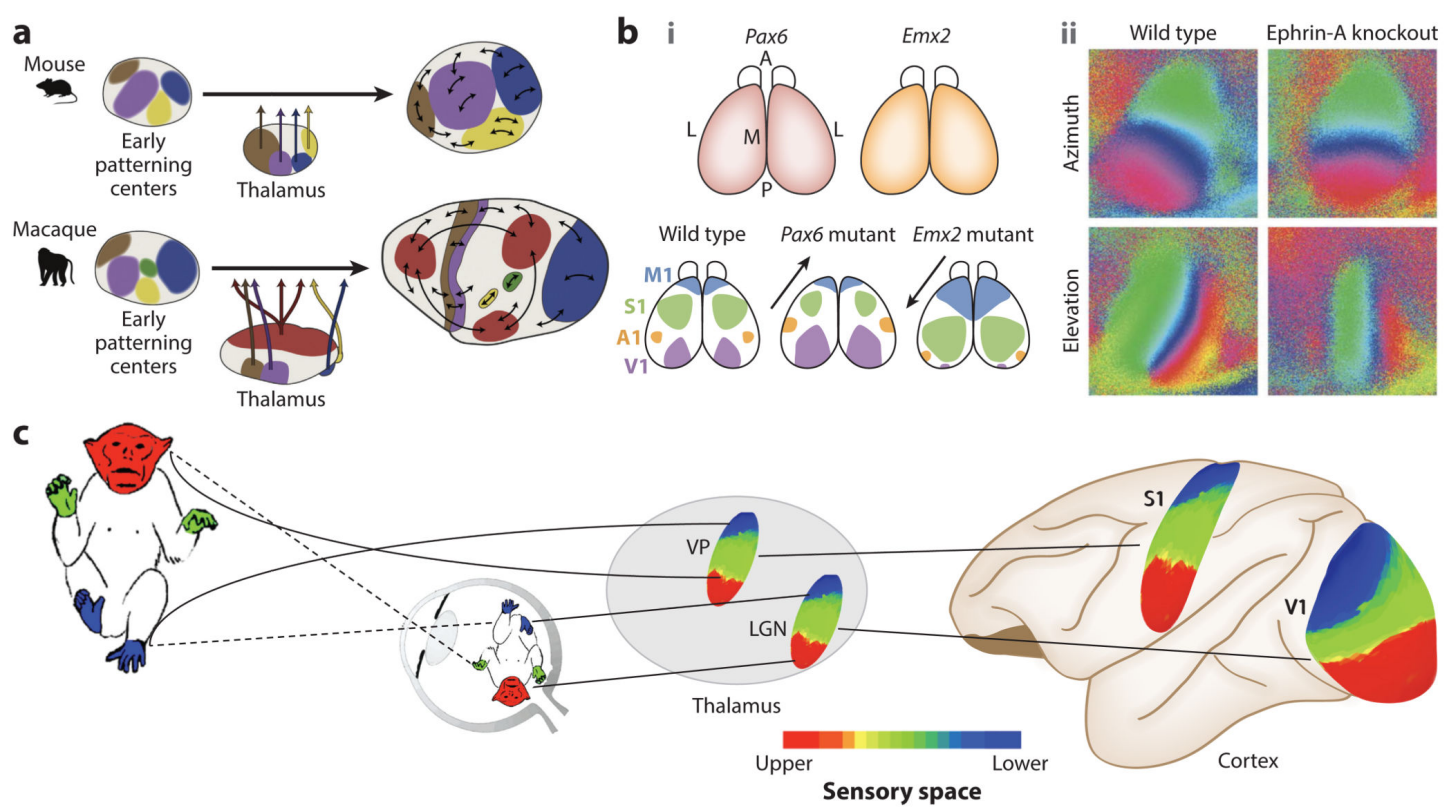
Map formation. (*a*) Evolutionarily preserved patterning of developing cortex. Panel *a* adapted with permission from Krienen & Buckner (2020). (*b*) Distortions in (*i*) areal patterning and (*ii*) internal map topography from changes in molecular guidance expression. Panel *b*, subpanel *i* adapted from Ochi et al. (2022) (CC BY 4.0); panel *b*, subpanel *ii* adapted with permission from Cang et al. (2005a). (*c*) Alignment of external-world orientation of sensory inputs across modalities. Abbreviations: LGN, lateral geniculate nucleus; S1, primary somatosensory cortex; V1, primary visual cortex; VP, ventral posterior nucleus.

The consistent positioning of sensory areas across mammals indicates that the areal organization across the cortical surface is evolutionarily old (Krubitzer 2007). Intrinsic genetic factors determine the topographic arrangement across cortex of primary areas that receive sensory input via the thalamus (McLaughlin & O’Leary 2005) (Figure 4). Gradients of expression of transcription factors, such as *Emx2*, *Pax6*, and other molecular markers, regulate the positioning of visual, motor, and auditory cortices and shape the entire cortical layout. Beyond areal patterning, these processes also lay the foundation for map topography within each primary sensory or motor area. For example, molecular gradients, such as those formed by ephrins, establish the axes for visual, olfactory, somatosensory, auditory, and motor maps (Flanagan 2006). Variations in expression of these genes distort topographies across modalities. Prenatal activity-dependent processes, such as retinal waves, refine these initial maps that are laid down by genetic factors (McLaughlin et al. 2003).

#### 4.1.3. Cortical map replication.

**Figure 5:**
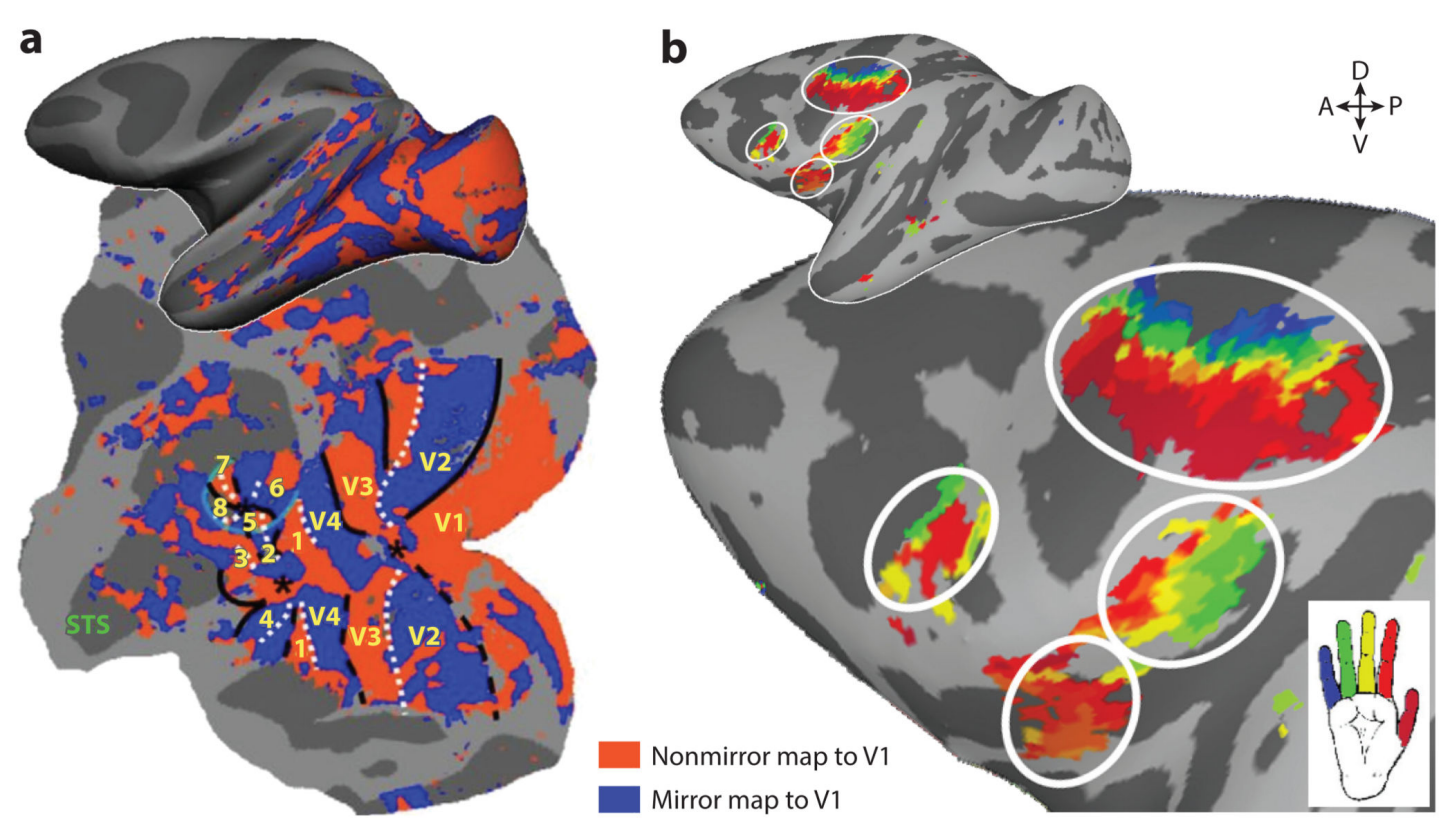
Mirror maps in visual and somatomotor cortices. (*a*) Red and blue indicate reversals in the topographic progression of visual space. Panel *a* adapted from Janssens et al. (2014) (CC BY-NC-SA 3.0). (*b*) Colors indicate the topographic progression of finger representations in several maps (*p <* 0.0001, uncorrected, for the digit representation with the largest beta value). Panel *b* adapted from Arcaro et al. (2019a). Abbreviations: A, anterior; D, dorsal; P, posterior; STS, superior temporal sulcus; V, ventral; V1–4, visual cortices.

Topographic maps of primary sensory areas replicate into secondary areas throughout cortex. Although the mechanisms for postprimary cortical development are less understood, we suggest that these areas also self-organize through activity-dependent interactions (Imam & Finlay 2020, Rosa 2002). Primary sensory areas project to adjacent cortex in a mirror-image fashion. Secondary areas in turn project in mirror-image fashion to the next adjacent cortical areas that project to further cortex. Self-organizing synaptic sorting leads to smooth continuous maps, explaining why cortical regions share map coordinates at borders and therefore form a mirror topographic map of their antecedent cortex. The consistent mirroring, coupled with the increasing complexity of neuronal tuning, indicates that higher areas develop by iterative rules rather than area-specific mechanisms (Figure 5). Other factors, including spatiotemporal patterns of guidance molecule expression (Barber et al. 2015) and nonprimary thalamic input (Martini et al. 2021), might further shape the development of nonprimary areas. The role of thalamic association nuclei is particularly notable as they connect topographically to cortex and establish connections around the same time that sensory thalamic nuclei first innervate primary cortex (Shatz & Rakic 1981). Thus, sensory areas beyond primary cortices are predisposed to topographic wiring. At a larger scale, this smoothness principle can lead to hierarchically structured functional streams (Aflalo & Graziano 2011).

Mammalian brain evolution is characterized by increasing cortical territory interspersed between primary sensory areas (Krubitzer 2007). Within a species, individuals typically have a uniform arrangement and number of maps across cortex. We do not know whether the number of these maps is predetermined, or whether map mirroring just iterates until the cortex is fully occupied. The fact that much of cortex consists of continuous mirror maps present at birth (Arcaro & Livingstone 2017a) indicates that a standardized cortical layout for each species requires only a predetermination of the primary zones and is then filled in by self-organizing mirrored maps.

#### 4.1.4. Topographic maps as a ubiquitous principle of cognitive systems.

Despite the prevalence of maps, there is a widespread notion that topography must not be suitable for information processing outside of sensory systems (Pylyshyn 1984). Yet, topographic organization exists throughout frontal and parietal association cortices (Silver & Kastner 2009). These maps typically respond only to cognitively demanding manipulations of spatial coding that engage attention and memory processes (Mackey et al. 2017). Beyond visuospatial representations, association cortex contains maps representing abstract cognitive dimensions such as physical quantities or event duration (Protopapa et al. 2019), object size (Harvey et al. 2015), or numerosity (Harvey et al. 2013). Beyond tonotopy, auditory and language areas are also topographically organized by temporal structure (Lerner et al. 2011). While usually driven by a single sensory modality, areas of overlap between maps could function as hubs of supramodal representations, integrating across modalities and abstracting features and processes.

### 4.2. An Interconnected Topographic Brain

In individuals with sensory loss, intact modalities can co-opt circuits normally dedicated to processing the impaired modality. For example, auditory stimulation in congenitally blind individuals activates visual cortex (Bedny et al. 2011). Several fMRI studies on congenitally blind subjects have focused attention on the nature of category domains in IT.

#### 4.2.1. Cross-modal mapping.

Blind individuals, in response to tactile or auditory stimulation related to faces (Ratan Murty et al. 2020, van den Hurk et al. 2017) or reading (Reich et al. 2011, Striem-Amit et al. 2012), activate areas analogous to those activated by visual stimulation in sighted individuals. Similarly, touching objects (Pietrini et al. 2004), tactile representations of scenes (Wolbers et al. 2011), spoken words for tools (Mahon et al. 2010), and spoken words for large versus small objects (He et al. 2013) all activate similar locations in blind subjects as visual presentation of objects, scenes, tools, or large versus small objects do in sighted subjects. These findings have been interpreted as meaning that VTC domains are innately predetermined to process that specific category because these activations cross sensory modalities. Given that IT receives predominantly visual inputs and is not inherently multimodal, the inference is that this cross-modal specificity must originate in higher association cortices. Thus, according to this account, cross-modal selectivity for the same category necessitates top-down influences to route information between sensory pathways.

#### 4.2.2. Map-preserving connectivity across modalities.

Contrary to this top-down account, we argue that cortical evolution and development are more consistent with a bottom-up, map-based mechanism. Higher cortical areas evolved later than early areas. Our evolutionary ancestors possessed less association cortex yet exhibited multimodal behaviors, such as looking toward a sound, even at birth. Moreover, feedforward inputs mature before their feedback counterparts (Burkhalter 1993), and the maturation of feedback connections is contingent on feedforward inputs (Ibrahim LA 2021). These observations underscore the central role that input pathways from the sensory periphery to cortex play during early development. These pathways, topographically organized at birth, dictate how sensory input is distributed across cortex. Importantly, this topographic structure persists even in individuals with loss of a sensory modality, as in the retinotopic organization in blind individuals (Butt et al. 2013) or the tonotopic organization in deaf subjects (Striem-Amit et al. 2016). Thus, even in cases where cross-modal activations are present, the topography within sensory cortices, and thereby the pathways of interareal communication, remains intact.

**Figure 6:**
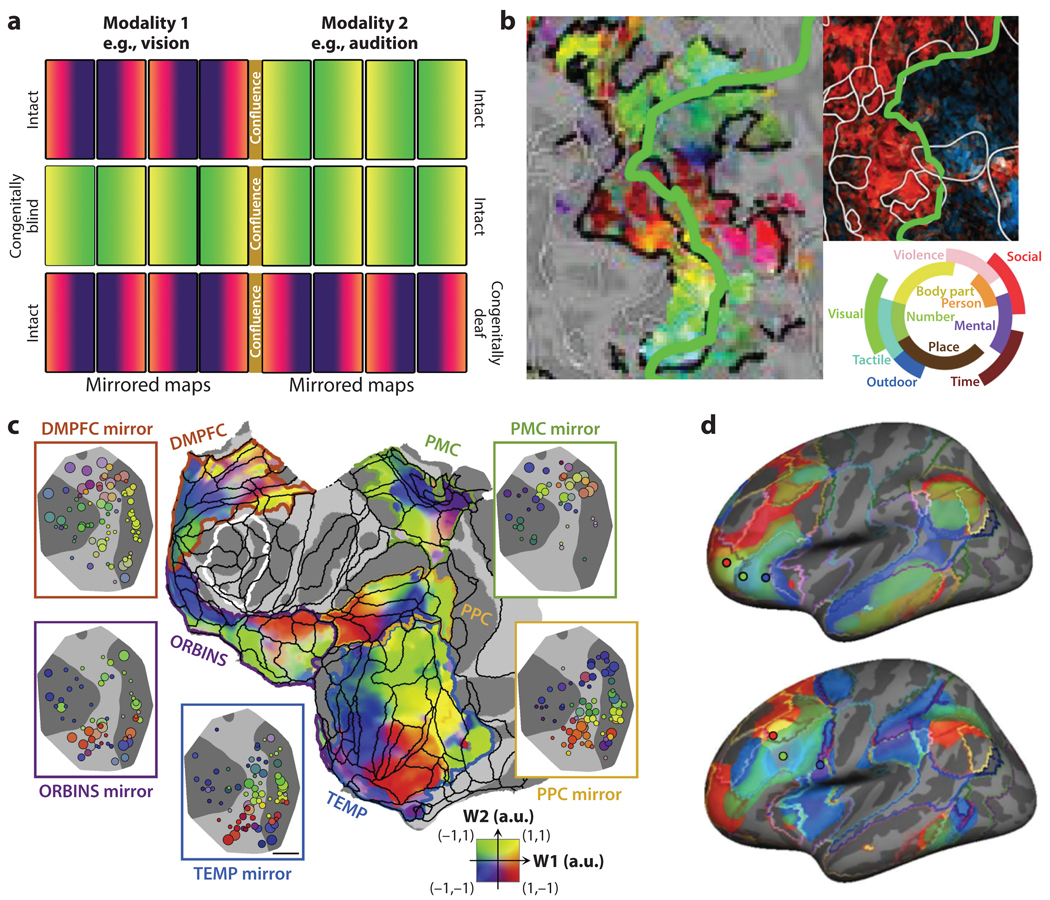
Principles of cross-modal map alignment. (*a*) Illustration of map convergence across modalities and takeover of deprived modality by intact modality. (*b*) Alignment of semantic categories in human lateral occipitotemporal cortex across visual-to-auditory modalities. The smaller image differentiates visual (*red*) from auditory (*blue*) activations. The green line illustrates the boundary between modalities. Panel *b* adapted with permission from Popham et al. (2021). (*c*) Topographic connectivity in macaque prefrontal cortex. Colors indicate corresponding points in topographic mapping between prefrontal and association cortices (frontal, parietal, and temporal). Panel *c* adapted with permission from Xu et al. (2022). (*d*) Topographic correlations in human prefrontal cortex. Each map shows three seeds (*red*, *green*, *blue*) in adjacent parts of prefrontal cortex, with correspondingly colored connectivity in temporal and parietal cortices. Panel *d* adapted from Yeo et al. (2011). Abbreviations: DMPFC, dorsomedial prefrontal cortex; ORBINS, orbitofrontal and insular cortices; PMC, posteromedial cortex; PPC, posterior parietal cortex; TEMP, temporal cortex.

We propose that the intrinsic topographies within modalities provide a parsimonious account for the alignment of maps across modalities. A substantial portion of the primate brain consists of maps of the external environment across different sensory modalities, even at birth (Figure 3a). Governed by shared developmental gradients (Flanagan 2006, O’Leary et al. 1999, Tessier-Lavigne & Goodman 1996), these maps lead to consistent topographic organization of external-world sensory space. This includes inversions of sensory space for both the visual field and body representations (upwards in the world is generally ventral in the brain, and down in the world is dorsal) and expanded high-resolution regions—central visual field in the visual system, fingertips in somatosensation, and fineness of motor control. Self-organizing mechanisms promote the mirroring of primary maps that spread out and occupy cortex in between primary maps. The coalignment of primary maps coupled with their replication could result in confluence zones where maps across modalities meet (Figure 6a). A globally aligned brain organization reduces the daunting challenge of connecting disparate points in a high-dimensional space, transforming it into the more tractable task of linking aligned low-dimensional maps. The inherent congruency of these topographic maps ensures a regularity of interlinked sensory modalities, as well as a mechanism for interacting with the environment and allowing spatially appropriate behaviors as early as birth.

Even without neurological impairments, congruency between sensory maps is evident, particularly in multimodal association cortex: In parietal cortex, individual neurons can process both somatosensory and visual input with aligned visual and somatosensory receptive fields (Duhamel et al. 1998). Temporal neurons can process both visual and auditory input, aligning sound localization with visual field location (Bruce et al. 1981, Hikosaka et al. 1988). Map congruency extends beyond sensory maps. In a human fMRI study looking at activity in response to video and audio stories (Popham et al. 2021) (Figure 6b), category specificity extended across the border from high-level visually responsive regions into high-level auditory/language regions (see the left side of Figure 6b), indicating that vision and language share a large-scale category topography. Similarly, another human fMRI study reported alignment of scene-perception and spatial-memory maps (Steel et al. 2021) (see the right side of Figure 6b). Congruency across modalities is likely further facilitated through topographic connectivity with frontal cortex (Xu et al. 2022, Yeo et al. 2011) (Figure 6c,d) and subcortically in the alignment of visual, somatosensory, and auditory representations across layers of the superior colliculus (Triplett et al. 2012). Such linking of sensory maps facilitates the processing of information about the same locations in the environment across different sensory modalities. When one sensory modality is impaired, these interconnections could route information from intact modalities to the affected-modality cortex.

The capacity for cross-modal mappings is contingent on early experience. Human fMRI studies reveal that early-onset sensory loss is pivotal for cross-modal reorganization (Bedny et al. 2012, Collignon et al. 2013). Consequently, the timing and nature of experiences within intact modalities also influence the capacity for reorganization (Badde et al. 2020). Electrophysiological recordings in cats indicate that early sensory input is essential for multisensory integration within the superior colliculus, suggesting that cross-modal mappings in intact brains are shaped by early experience (Wallace et al. 2004).

#### 4.2.3. Ubiquity of maps across modalities.

In the VTC, isoeccentricity representations are broadly aligned anteroposteriorly, varying in eccentricity from lateral to medial cortex. We speculate that, projecting that alignment via either convergence of maps across modalities or long-range connections with association cortex, the central visual field aligns through intermediary association cortices with low frequencies in the auditory map and, eventually, with the face part of the somatosensory and motor body maps. It would follow that central visual field representations, which are biased by scale and by looking behavior to become specialized during development for processing faces, would be, early in development, congruent with and interconnected with the low-frequency part of the auditory map [which itself becomes specialized for processing language (Moerel et al. 2012)] and with the face parts of the somatosensory map. Interestingly, natural sounds can be decoded along the eccentricity axis of early visual cortex in congenitally blind individuals (Vetter et al. 2020). This finding indicates that cross-modal reorganization might be primarily constrained by the topographic organization of not only the VTC but also the entire cortex. Thus, activations in blind individuals during Braille character recognition (Bola et al. 2019, Sadato et al. 1996) and localization of sound space (Campus et al. 2017, Norman & Thaler 2019) within foveal parts of early visual cortex might be better understood as an alignment of high-resolution spatial processing rather than as a remapping of specific functionalities. In this framework, cross-modal plasticity indicates commonalities in general computational requirements rather than commonalities in what the computations are used for.

We further propose that even higher association areas inherit these topographies. For example, we speculate that visual domains for faces, scenes, colors, and depth even in frontal cortex may be organized topographically, reflecting topography inherited from IT (Xu et al. 2022). Thus, the convergence of inputs from early visual retinotopic maps that lead to topographic organization of experienced categories in mid-level areas such as IT or ventral occipital cortex could also lead to still more abstract and multimodal topographies in higher areas outside of visual cortex, such as prefrontal cortex. In this framework, the sensory input pathways drive the specializations in amodal association areas, not the other way around as proposed by the top-down perspective.

## 5. CONCLUSION: ARE EVEN HIGHER COGNITIVE FUNCTIONS, SUCH AS LANGUAGE, TOPOGRAPHIC?

Lastly, we ask whether the concept of activity acting on maps can be extended to more complex cognitive systems, such as language. The debate over the nature of language, and specifically the claim of a universal grammar, parallels the fervent discussions surrounding the nature of visual domains in temporal cortex. Both debates probe the core of human cognition, seeking to understand the interplay between our innate abilities and the experiences that shape them.

Because language is universally and exclusively human, it is widely believed that humans, uniquely, possess brain structures that allow, even compel, us to learn, understand, and produce language and that these circuits evolved specifically for language because of its adaptive benefits. Chomsky (1965) influentially argued that language must be innate: First, the ease with which children learn language suggests that humans must have a genetically predetermined part of their brain that evolved specifically for language. Second, because the learning set (the sentences children hear) is finite, they cannot have learned all the sentences they could possibly utter, yet they utter novel grammatical sentences. Third, human languages share arbitrary grammatical features that represent only a fraction of all possible grammars. However subsequent linguistic research has challenged many of these proposed universal rules, making it unclear what exactly is universal about language (Everett 2005, Goldberg 2008, Hauser et al. 2002). The argument for language’s innateness faces further challenges when considering the evolutionary timeline. The emergence of language coincides with human migration out of Africa. If a dedicated language module evolved, then why did such genes appear so abruptly and then seemingly stagnate in their evolutionary trajectory? As humans migrated, evolution tailored human physical features to specific environments: skin pigmentation adjusted for sun exposure, facial features adapted to varying climates, and lactose tolerance correlated with domestication of milk-producing animals. If language-specific genes existed, they too should have continued to evolve and adapt to local linguistic environments, resulting in a spectrum of biologically encoded universal grammars (Lieberman 2013). Yet, there is no evidence that individuals are predisposed to learn languages aligned with their genetic lineage. Instead of viewing the brain’s language circuitry as a genetic adaptation, it might be that languages evolved to fit our brain structures. Children’s ease of learning language might stem not from innate, language-specific circuitry but because languages themselves are a cultural adaptation that evolved to be easily learned and processed by the domain-general architecture of our brains. Recent computational modelling demonstrates that grammatical structures can emerge from domain-general architectures (Goldstein et al. 2022), which raises the question of what, if any, language-specific core knowledge is built into the brain.

Adult humans have distinct brain regions specifically involved in producing and processing language; damage to these regions impairs language abilities. However, the existence of specialized language domains does not necessarily imply that they are innately specialized. Humans also have distinct brain regions specialized in processing faces, and damage to these regions results in face recognition deficits, but experience is required for the development of face domains. Similarly, literate adults have text-specific brain regions, and damage to those regions impairs reading, yet text domains do not emerge unless an individual has learned to read. Given literacy’s relatively recent emergence in human history, it is impossible that text recognition is innate. Thus, we argue that, just as face recognition is not innate, and reading clearly is not, language also is not innate. Instead, we propose that language-specific circuits develop during childhood as a consequence of extensive early exposure to language. Neuroimaging studies show that language-specific architecture emerges gradually postnatally (Kujala et al. 2023). While the neonate brain already shows differential responses to forward and backward speech (Pena et al. 2003), we posit that this reflects a neonate’s ability to learn and differentiate the statistical structure of their experienced speech (Saffran et al. 1996, Teinonen et al. 2009). Another parallel can be drawn between the development of language, face recognition, and other domains that endow expertise: They are best learned during childhood. People who learn a new language after puberty rarely have fluency comparable to that of childhood learners. Similarly, our research indicates that juvenile monkeys are better at learning to recognize symbols than are adults. While it remains uncertain whether people who learn to read as adults are less fluent than those who learned in childhood, we think it is likely they would be.

In sum, this perspective highlights the dynamic, adaptable, and resilient nature of human cognition, positing that our expertise and abilities are largely sculpted by experience constrained by a topographic brain architecture.

## References

[R1] Aflalo TN, Graziano MSA. 2011. Organization of the macaque extrastriate visual cortex re-examined using the principle of spatial continuity of function. J. Neurophysiol 105:305–20 21068269 10.1152/jn.00795.2010 PMC3023372

[R2] Arcaro M 2023. The building blocks of vision: evidence for a hierarchical, retinotopic organization in the human neonate brain. J. Vis 23:5535

[R3] Arcaro M, Schade P, Livingstone M. 2018. Preserved cortical organization in the absence of early visual input. J. Vis 18:27

[R4] Arcaro MJ, Livingstone MS. 2017a. A hierarchical, retinotopic proto-organization of the primate visual system at birth. eLife 6:e26196 10.7554/eLife.26196 PMC5495573 28671063

[R5] Arcaro MJ, Livingstone MS. 2017b. Retinotopic organization of scene areas in macaque inferior temporal cortex. J. Neurosci 37:7373–89 28674177 10.1523/JNEUROSCI.0569-17.2017 PMC5546109

[R6] Arcaro MJ, Livingstone MS. 2021. On the relationship between maps and domains in inferotemporal cortex. Nat. Rev. Neurosci 22:573–83 34345018 10.1038/s41583-021-00490-4 PMC8865285

[R7] Arcaro MJ, Livingstone MS, Kay KN, Weiner KS. 2022. The retrocalcarine sulcus maps different retinotopic representations in macaques and humans. Brain Struct. Funct 227:1227–45 34921348 10.1007/s00429-021-02427-0 PMC9046316

[R8] Arcaro MJ, McMains SA, Singer BD, Kastner S. 2009. Retinotopic organization of human ventral visual cortex. J. Neurosci 29:10638–52 19710316 10.1523/JNEUROSCI.2807-09.2009 PMC2775458

[R9] Arcaro MJ, Schade PF, Livingstone MS. 2019a. Body map proto-organization in newborn macaques. PNAS 116:24861–71 31732670 10.1073/pnas.1912636116 PMC6900594

[R10] Arcaro MJ, Schade PF, Livingstone MS. 2019b. Universal mechanisms and the development of the face network: What you see is what you get. Annu. Rev. Vis. Sci 5:341–72 31226011 10.1146/annurev-vision-091718-014917 PMC7568401

[R11] Arcaro MJ, Schade PF, Vincent JL, Ponce CR, Livingstone MS. 2017. Seeing faces is necessary for face-domain formation. Nat. Neurosci 20:1404–12 28869581 10.1038/nn.4635 PMC5679243

[R12] Attneave F 1954. Some informational aspects of visual perception. Psychol. Rev 61:183–93 13167245 10.1037/h0054663

[R13] Badde S, Ley P, Rajendran SS, Shareef I, Kekunnaya R, Roder B. 2020. Sensory experience during early sensitive periods shapes cross-modal temporal biases. eLife 9:e1238 10.7554/eLife.61238 PMC7476755 32840213

[R14] Baek S, Song M, Jang J, Kim G, Paik SB. 2021. Face detection in untrained deep neural networks. Nat. Commun 12:7328 34916514 10.1038/s41467-021-27606-9 PMC8677765

[R15] Baldwin MK, Kaskan PM, Zhang B, Chino YM, Kaas JH. 2012. Cortical and subcortical connections of V1 and V2 in early postnatal macaque monkeys. J. Comp. Neurol 520:544–69 21800316 10.1002/cne.22732 PMC3673566

[R16] Bao P, She L, McGill M, Tsao DY. 2020. A map of object space in primate inferotemporal cortex. Nature 583:103–8 32494012 10.1038/s41586-020-2350-5 PMC8088388

[R17] Barber M, Arai Y, Morishita Y, Vigier L, Causeret F, 2015. Migration speed of Cajal-Retzius cells modulated by vesicular trafficking controls the size of higher-order cortical areas. Curr. Biol 25:2466–78 26387718 10.1016/j.cub.2015.08.028

[R18] Batardiere A, Barone P, Knoblauch K, Giroud P, Berland M, 2002. Early specification of the hierarchical organization of visual cortical areas in the macaque monkey. Cereb. Cortex 12:453–65 11950763 10.1093/cercor/12.5.453

[R19] Bedny M, Pascual-Leone A, Dodell-Feder D, Fedorenko E, Saxe R. 2011. Language processing in the occipital cortex of congenitally blind adults. PNAS 108:4429–34 21368161 10.1073/pnas.1014818108 PMC3060248

[R20] Bedny M, Pascual-Leone A, Dravida S, Saxe R. 2012. A sensitive period for language in the visual cortex: distinct patterns of plasticity in congenitally versus late blind adults. Brain Lang. 122:162–70 22154509 10.1016/j.bandl.2011.10.005 PMC3536016

[R21] Bishop KM, Rubenstein JLR, O’Leary DDM. 2002. Distinct actions of *Emx1*, *Emx2*, and *Pax6* in regulating the specification of areas in the developing neocortex. J. Neurosci 22:7627–38 12196586 10.1523/JNEUROSCI.22-17-07627.2002 PMC6757966

[R22] Blauch NM, Behrmann M, Plaut DC. 2022. A connectivity-constrained computational account of topographic organization in primate high-level visual cortex. PNAS 119:e2112566119 10.1073/pnas.2112566119 PMC8784138 35027449

[R23] Bola L, Matuszewski J, Szczepanik M, Drozdziel D, Sliwinska MW, 2019. Functional hierarchy for tactile processing in the visual cortex of sighted adults. NeuroImage 202:116084 10.1016/j.neuroimage.2019.116084 31400530

[R24] Bruce C, Desimone R, Gross CG. 1981. Visual properties of neurons in a polysensory area in superior temporal sulcus of the macaque. J. Neurophysiol 46:369–84 6267219 10.1152/jn.1981.46.2.369

[R25] Burkhalter A 1993. Development of forward and feedback connections between areas V1 and V2 of human visual cortex. Cereb. Cortex 3:476–87 8260814 10.1093/cercor/3.5.476

[R26] Bushnell IWR. 2001. Mother’s face recognition in newborn infants: learning and memory. Infant Child Dev. 10:67–74

[R27] Butt OH, Benson NC, Datta R, Aguirre GK. 2013. The fine-scale functional correlation of striate cortex in sighted and blind people. J. Neurosci 33:16209–19 24107953 10.1523/JNEUROSCI.0363-13.2013 PMC3792460

[R28] Campus C, Sandini G, Concetta Morrone M, Gori M. 2017. Spatial localization of sound elicits early responses from occipital visual cortex in humans. Sci. Rep 7:10415 28874681 10.1038/s41598-017-09142-z PMC5585168

[R29] Cang J, Kaneko M, Yamada J, Woods G, Stryker MP, Feldheim DA. 2005a. Ephrin-As guide the formation of functional maps in the visual cortex. Neuron 48:577–89 16301175 10.1016/j.neuron.2005.10.026 PMC2424263

[R30] Cang J, Renteria RC, Kaneko M, Liu X, Copenhagen DR, Stryker MP. 2005b. Development of precise maps in visual cortex requires patterned spontaneous activity in the retina. Neuron 48:797–809 16337917 10.1016/j.neuron.2005.09.015 PMC2562716

[R31] Chino Y, Smith EL 3rd, Zhang B, Matsuura K, Mori T, Kaas JH. 2001. Recovery of binocular responses by cortical neurons after early monocular lesions. Nat. Neurosci 4:689–90 11426222 10.1038/89469

[R32] Chomsky N 1965. Aspects of the Theory of Syntax. Cambridge, MA: MIT Press

[R33] Coggan DD, Tong F. 2023. Spikiness and animacy as potential organizing principles of human ventral visual cortex. Cereb. Cortex 33:8194–217 36958809 10.1093/cercor/bhad108 PMC10321104

[R34] Cohen L, Dehaene S, Naccache L, Lehericy S, Dehaene-Lambertz G, 2000. The visual word form area: spatial and temporal characterization of an initial stage of reading in normal subjects and posterior split-brain patients. Brain 123(Pt. 2):291–307 10648437 10.1093/brain/123.2.291

[R35] Collignon O, Dormal G, Albouy G, Vandewalle G, Voss P, 2013. Impact of blindness onset on the functional organization and the connectivity of the occipital cortex. Brain 136:2769–83 23831614 10.1093/brain/awt176

[R36] Dall’Orso S, Steinweg J, Allievi AG, Edwards AD, Burdet E, Arichi T. 2018. Somatotopic mapping of the developing sensorimotor cortex in the preterm human brain. Cereb. Cortex 28:2507–15 29901788 10.1093/cercor/bhy050 PMC5998947

[R37] Deen B, Richardson H, Dilks DD, Takahashi A, Keil B, 2017. Organization of high-level visual cortex in human infants. Nat. Commun 8:13995 28072399 10.1038/ncomms13995 PMC5234071

[R38] Dehaene S, Pegado F, Braga LW, Ventura P, Filho GN, 2010. How learning to read changes the cortical networks for vision and language. Science 330:1359–64 21071632 10.1126/science.1194140

[R39] Distler C, Bachevalier J, Kennedy C, Mishkin M, Ungerleider LG. 1996. Functional development of the corticocortical pathway for motion analysis in the macaque monkey: a ^14^C-2-deoxyglucose study. Cereb. Cortex 6:184–95 8670649 10.1093/cercor/6.2.184

[R40] Downing PE, Chan AW, Peelen MV, Dodds CM, Kanwisher N. 2006. Domain specificity in visual cortex. Cereb. Cortex 16:1453–61 16339084 10.1093/cercor/bhj086

[R41] Duhamel JR, Colby CL, Goldberg ME. 1998. Ventral intraparietal area of the macaque: congruent visual and somatic response properties. J. Neurophysiol 79:126–36 9425183 10.1152/jn.1998.79.1.126

[R42] Everett DL. 2005. Cultural constraints on grammar and cognition in Pirahã: another look at the design features of human language. Curr. Anthropol 46:621–34

[R43] Feng X, Monzalvo K, Dehaene S, Dehaene-Lambertz G. 2022. Evolution of reading and face circuits during the first three years of reading acquisition. NeuroImage 259:119394 10.1016/j.neuroimage.2022.119394 35718022

[R44] Flanagan JG. 2006. Neural map specification by gradients. Curr. Opin. Neurobiol 16:59–66 16417998 10.1016/j.conb.2006.01.010

[R45] Golarai G, Ghahremani DG, Greenwood AC, Gabrieli JDE, Eberhardt JL. 2021. The development of race effects in face processing from childhood through adulthood: neural and behavioral evidence. Dev. Sci 24:e13058 10.1111/desc.13058 33151616

[R46] Golarai G, Ghahremani DG, Whitfield-Gabrieli S, Reiss A, Eberhardt JL, 2007. Differential development of high-level visual cortex correlates with category-specific recognition memory. Nat. Neurosci 10:512–22 17351637 10.1038/nn1865 PMC3660101

[R47] Goldberg AE. 2008. Universal grammar? Or prerequisites for natural language? Behav. Brain Sci 31:522–23

[R48] Goldstein A, Zada Z, Buchnik E, Schain M, Price A, 2022. Shared computational principles for language processing in humans and deep language models. Nat. Neurosci 25:369–80 35260860 10.1038/s41593-022-01026-4 PMC8904253

[R49] Gomez J, Barnett M, Grill-Spector K. 2019. Extensive childhood experience with Pokémon suggests eccentricity drives organization of visual cortex. Nat. Hum. Behav 3:611–24 31061489 10.1038/s41562-019-0592-8 PMC7055538

[R50] Gomez J, Natu V, Jeska B, Barnett M, Grill-Spector K. 2018. Development differentially sculpts receptive fields across early and high-level human visual cortex. Nat. Commun 9:788 29476135 10.1038/s41467-018-03166-3 PMC5824941

[R51] Grill-Spector K, Weiner KS. 2014. The functional architecture of the ventral temporal cortex and its role in categorization. Nat. Rev. Neurosci 15:536–48 24962370 10.1038/nrn3747 PMC4143420

[R52] Harvey BM, Fracasso A, Petridou N, Dumoulin SO. 2015. Topographic representations of object size and relationships with numerosity reveal generalized quantity processing in human parietal cortex. PNAS 112:13525–30 26483452 10.1073/pnas.1515414112 PMC4640722

[R53] Harvey BM, Klein BP, Petridou N, Dumoulin SO. 2013. Topographic representation of numerosity in the human parietal cortex. Science 341:1123–26 24009396 10.1126/science.1239052

[R54] Hasson U, Levy I, Behrmann M, Hendler T, Malach R. 2002. Eccentricity bias as an organizing principle for human high-order object areas. Neuron 34:479–90 11988177 10.1016/s0896-6273(02)00662-1

[R55] Hauser MD, Chomsky N, Fitch WT. 2002. The faculty of language: What is it, who has it, and how did it evolve? Science 298:1569–79 12446899 10.1126/science.298.5598.1569

[R56] He C, Peelen MV, Han Z, Lin N, Caramazza A, Bi Y. 2013. Selectivity for large nonmanipulable objects in scene-selective visual cortex does not require visual experience. NeuroImage 79:1–9 23624496 10.1016/j.neuroimage.2013.04.051

[R57] Hikosaka K, Iwai E, Saito H, Tanaka K. 1988. Polysensory properties of neurons in the anterior bank of the caudal superior temporal sulcus of the macaque monkey. J. Neurophysiol 60:1615–37 2462027 10.1152/jn.1988.60.5.1615

[R58] Hubel DH, Livingstone MS. 1987. Segregation of form, color, and stereopsis in primate area 18. J. Neurosci 7:3378–415 2824714 10.1523/JNEUROSCI.07-11-03378.1987 PMC6569042

[R59] Hubel DH, Wiesel TN. 1965. Receptive fields and functional architecture in two nonstriate visual areas (18 and 19) of the cat. J. Neurophysiol 28:229–89 14283058 10.1152/jn.1965.28.2.229

[R60] Ibrahim LA, Huang S, Fernandez-Otero M, Sherer M, Qiu Y, 2021. Bottom-up inputs are required for establishment of top-down connectivity onto cortical layer 1 neurogliaform cells. Neuron 109:3473–85 34478630 10.1016/j.neuron.2021.08.004 PMC9316418

[R61] Imam N, Finlay B. 2020. Self-organization of cortical areas in the development and evolution of neocortex. PNAS 117:29212–20 33139564 10.1073/pnas.2011724117 PMC7682404

[R62] Janssens T, Zhu Q, Popivanov ID, Vanduffel W. 2014. Probabilistic and single-subject retinotopic maps reveal the topographic organization of face patches in the macaque cortex. J. Neurosci 34:10156–67 25080579 10.1523/JNEUROSCI.2914-13.2013 PMC6608270

[R63] Jayaraman S, Fausey CM, Smith LB. 2017. Why are faces denser in the visual experiences of younger than older infants? Dev. Psychol 53:38–49 28026190 10.1037/dev0000230 PMC5271576

[R64] Kaas JH. 1997. Topographic maps are fundamental to sensory processing. Brain Res. Bull 44:107–12 9292198 10.1016/s0361-9230(97)00094-4

[R65] Kamps FS, Hendrix CL, Brennan PA, Dilks DD. 2020. Connectivity at the origins of domain specificity in the cortical face and place networks. PNAS 117:6163–69 32123077 10.1073/pnas.1911359117 PMC7084100

[R66] Kolster H, Janssens T, Orban GA, Vanduffel W. 2014. The retinotopic organization of macaque occipitotemporal cortex anterior to V4 and caudoventral to the middle temporal (MT) cluster. J. Neurosci 34:10168–91 25080580 10.1523/JNEUROSCI.3288-13.2014 PMC4115132

[R67] Kolster H, Peeters R, Orban GA. 2010. The retinotopic organization of the human middle temporal area MT/V5 and its cortical neighbors. J. Neurosci 30:9801–20 20660263 10.1523/JNEUROSCI.2069-10.2010 PMC6632824

[R68] Konkle T, Caramazza A. 2013. Tripartite organization of the ventral stream by animacy and object size. J. Neurosci 33:10235–42 23785139 10.1523/JNEUROSCI.0983-13.2013 PMC3755177

[R69] Konkle T, Oliva A. 2012. A real-world size organization of object responses in occipitotemporal cortex. Neuron 74:1114–24 22726840 10.1016/j.neuron.2012.04.036 PMC3391318

[R70] Kosakowski HL, Cohen MA, Takahashi A, Keil B, Kanwisher N, Saxe R. 2022. Selective responses to faces, scenes, and bodies in the ventral visual pathway of infants. Curr. Biol 32:265–74.e5 34784506 10.1016/j.cub.2021.10.064 PMC8792213

[R71] Krienen FM, Buckner RL. 2020. Human association cortex: expanded, untethered, neotenous, and plastic. In Evolutionary Neuroscience, ed. Kaas JH, pp. 845–60. London: Academic. 2nd ed.

[R72] Krubitzer L 2007. The magnificent compromise: cortical field evolution in mammals. Neuron 56:201–8 17964240 10.1016/j.neuron.2007.10.002

[R73] Kujala T, Partanen E, Virtala P, Winkler I. 2023. Prerequisites of language acquisition in the newborn brain. Trends Neurosci. 46:726–37 37344237 10.1016/j.tins.2023.05.011

[R74] Lafer-Sousa R, Conway BR. 2013. Parallel, multi-stage processing of colors, faces and shapes in macaque inferior temporal cortex. Nat. Neurosci 16:1870–78 24141314 10.1038/nn.3555 PMC3957328

[R75] Lafer-Sousa R, Conway BR, Kanwisher NG. 2016. Color-biased regions of the ventral visual pathway lie between face- and place-selective regions in humans, as in macaques. J. Neurosci 36:1682–97 26843649 10.1523/JNEUROSCI.3164-15.2016 PMC4737777

[R76] Lerner Y, Honey CJ, Silbert LJ, Hasson U. 2011. Topographic mapping of a hierarchy of temporal receptive windows using a narrated story. J. Neurosci 31:2906–15 21414912 10.1523/JNEUROSCI.3684-10.2011 PMC3089381

[R77] Lieberman P 2013. The Unpredictable Species: What Makes Humans Unique. Princeton, NJ: Princeton Univ. Press

[R78] Livingstone MS, Vincent JL, Arcaro MJ, Srihasam K, Schade PF, Savage T. 2017. Development of the macaque face-patch system. Nat. Commun 8:14897 28361890 10.1038/ncomms14897 PMC5381009

[R79] Long B, Yu CP, Konkle T. 2018. Mid-level visual features underlie the high-level categorical organization of the ventral stream. PNAS 115:E9015–24 30171168 10.1073/pnas.1719616115 PMC6156638

[R80] Mackey WE, Winawer J, Curtis CE. 2017. Visual field map clusters in human frontoparietal cortex. eLife 6:e22974 10.7554/eLife.22974 PMC5491263 28628004

[R81] Mahon BZ, Schwarzbach J, Caramazza A. 2010. The representation of tools in left parietal cortex is independent of visual experience. Psychol. Sci 21:764–71 20483823 10.1177/0956797610370754 PMC2908275

[R82] Martini FJ, Guillamon-Vivancos T, Moreno-Juan V, Valdeolmillos M, Lopez-Bendito G. 2021. Spontaneous activity in developing thalamic and cortical sensory networks. Neuron 109:2519–34 34293296 10.1016/j.neuron.2021.06.026 PMC7611560

[R83] McLaughlin T, O’Leary DD. 2005. Molecular gradients and development of retinotopic maps. Annu. Rev. Neurosci 28:327–55 16022599 10.1146/annurev.neuro.28.061604.135714

[R84] McLaughlin T, Torborg CL, Feller MB, O’Leary DD. 2003. Retinotopic map refinement requires spontaneous retinal waves during a brief critical period of development. Neuron 40:1147–60 14687549 10.1016/s0896-6273(03)00790-6

[R85] Moerel M, De Martino F, Formisano E. 2012. Processing of natural sounds in human auditory cortex: tonotopy, spectral tuning, and relation to voice sensitivity. J. Neurosci 32:14205–16 23055490 10.1523/JNEUROSCI.1388-12.2012 PMC6622378

[R86] Mountcastle VB. 1957. Modality and topographic properties of single neurons of cat’s somatic sensory cortex. J. Neurophysiol 20:408–34 13439410 10.1152/jn.1957.20.4.408

[R87] Nasr S, Echavarria CE, Tootell RB. 2014. Thinking outside the box: rectilinear shapes selectively activate scene-selective cortex. J. Neurosci 34:6721–35 24828628 10.1523/JNEUROSCI.4802-13.2014 PMC4019792

[R88] Norman LJ, Thaler L. 2019. Retinotopic-like maps of spatial sound in primary ‘visual’ cortex of blind human echolocators. Proc. Biol. Sci 286:20191910 10.1098/rspb.2019.1910 PMC6790759 31575359

[R89] Ochi S, Manabe S, Kikkawa T, Osumi N. 2022. Thirty years’ history since the discovery of Pax6: from central nervous system development to neurodevelopmental disorders. Int. J. Mol. Sci 23:6115 35682795 10.3390/ijms23116115 PMC9181425

[R90] O’Leary DD, Yates PA, McLaughlin T. 1999. Molecular development of sensory maps: representing sights and smells in the brain. Cell 96:255–69 9988220 10.1016/s0092-8674(00)80565-6

[R91] Op de Beeck HP, Torfs K, Wagemans J. 2008. Perceived shape similarity among unfamiliar objects and the organization of the human object vision pathway. J. Neurosci 28:10111–23 18829969 10.1523/JNEUROSCI.2511-08.2008 PMC6671279

[R92] Pena M, Maki A, Kovacic D, Dehaene-Lambertz G, Koizumi H, 2003. Sounds and silence: an optical topography study of language recognition at birth. PNAS 100:11702–5 14500906 10.1073/pnas.1934290100 PMC208821

[R93] Pietrini P, Furey ML, Ricciardi E, Gobbini MI, Wu WH, 2004. Beyond sensory images: object-based representation in the human ventral pathway. PNAS 101:5658–63 15064396 10.1073/pnas.0400707101 PMC397466

[R94] Ponce CR, Hartmann TS, Livingstone MS. 2017. End-stopping predicts curvature tuning along the ventral stream. J. Neurosci 37:648–59 28100746 10.1523/JNEUROSCI.2507-16.2016 PMC5242411

[R95] Pons TP, Garraghty PE, Ommaya AK, Kaas JH, Taub E, Mishkin M. 1991. Massive cortical reorganization after sensory deafferentation in adult macaques. Science 252:1857–60 1843843 10.1126/science.1843843

[R96] Popham SF, Huth AG, Bilenko NY, Deniz F, Gao JS, 2021. Visual and linguistic semantic representations are aligned at the border of human visual cortex. Nat. Neurosci 24:1628–36 34711960 10.1038/s41593-021-00921-6

[R97] Protopapa F, Hayashi MJ, Kulashekhar S, van der Zwaag W, Battistella G, 2019. Chronotopic maps in human supplementary motor area. PLOS Biol. 17:e3000026 10.1371/journal.pbio.3000026 PMC6428248 30897088

[R98] Pylyshyn ZW. 1984. Computation and Cognition: Toward a Foundation for Cognitive Science. Cambridge, MA: MIT Press

[R99] Ratan Murty NA, Teng S, Beeler D, Mynick A, Oliva A, Kanwisher N. 2020. Visual experience is not necessary for the development of face-selectivity in the lateral fusiform gyrus. PNAS 117:23011–20 32839334 10.1073/pnas.2004607117 PMC7502773

[R100] Reich L, Szwed M, Cohen L, Amedi A. 2011. A ventral visual stream reading center independent of visual experience. Curr. Biol 21:363–68 21333539 10.1016/j.cub.2011.01.040

[R101] Rodman HR, Scalaidhe SP, Gross CG. 1993. Response properties of neurons in temporal cortical visual areas of infant monkeys. J. Neurophysiol 70:1115–36 8229162 10.1152/jn.1993.70.3.1115

[R102] Roe AW, Pallas SL, Hahm JO, Sur M. 1990. A map of visual space induced in primary auditory cortex. Science 250:818–20 2237432 10.1126/science.2237432

[R103] Rosa MG. 2002. Visual maps in the adult primate cerebral cortex: some implications for brain development and evolution. Braz. J. Med. Biol. Res 35:1485–98 12436190 10.1590/s0100-879x2002001200008

[R104] Sadato N, Pascual-Leone A, Grafman J, Ibanez V, Deiber MP, . 1996. Activation of the primary visual cortex by Braille reading in blind subjects. Nature 380:526–28 8606771 10.1038/380526a0

[R105] Saffran JR, Aslin RN, Newport EL. 1996. Statistical learning by 8-month-old infants. Science 274:1926–28 8943209 10.1126/science.274.5294.1926

[R106] Scherf KS, Behrmann M, Humphreys K, Luna B. 2007. Visual category-selectivity for faces, places and objects emerges along different developmental trajectories. Dev. Sci 10:F15–30 17552930 10.1111/j.1467-7687.2007.00595.x

[R107] Shatz CJ, Rakic P. 1981. The genesis of efferent connections from the visual cortex of the fetal rhesus monkey. J. Comp. Neurol 196:287–307 7217358 10.1002/cne.901960208

[R108] Silver MA, Kastner S. 2009. Topographic maps in human frontal and parietal cortex. Trends Cogn. Sci 13:488–95 19758835 10.1016/j.tics.2009.08.005 PMC2767426

[R109] Srihasam K, Mandeville JB, Morocz IA, Sullivan KJ, Livingstone MS. 2012. Behavioral and anatomical consequences of early versus late symbol training in macaques. Neuron 73:608–19 22325210 10.1016/j.neuron.2011.12.022 PMC3278713

[R110] Srihasam K, Vincent JL, Livingstone MS. 2014. Novel domain formation reveals proto-architecture in inferotemporal cortex. Nat. Neurosci 17:1776–83 25362472 10.1038/nn.3855 PMC4241119

[R111] Steel A, Billings MM, Silson EH, Robertson CE. 2021. A network linking scene perception and spatial memory systems in posterior cerebral cortex. Nat. Commun 12:2632 33976141 10.1038/s41467-021-22848-z PMC8113503

[R112] Striem-Amit E, Almeida J, Belledonne M, Chen Q, Fang Y, 2016. Topographical functional connectivity patterns exist in the congenitally, prelingually deaf. Sci. Rep 6:29375 27427158 10.1038/srep29375 PMC4947901

[R113] Striem-Amit E, Cohen L, Dehaene S, Amedi A. 2012. Reading with sounds: sensory substitution selectively activates the visual word form area in the blind. Neuron 76:640–52 23141074 10.1016/j.neuron.2012.08.026

[R114] Striem-Amit E, Ovadia-Caro S, Caramazza A, Margulies DS, Villringer A, Amedi A. 2015. Functional connectivity of visual cortex in the blind follows retinotopic organization principles. Brain 138:1679–95 25869851 10.1093/brain/awv083 PMC4614142

[R115] Sugita Y 2008. Face perception in monkeys reared with no exposure to faces. PNAS 105:394–98 18172214 10.1073/pnas.0706079105 PMC2224224

[R116] Teinonen T, Fellman V, Näätänen R, Alku P, Huotilainen M. 2009. Statistical language learning in neonates revealed by event-related brain potentials. BMC Neurosci. 10:21 19284661 10.1186/1471-2202-10-21 PMC2670827

[R117] Tessier-Lavigne M, Goodman CS. 1996. The molecular biology of axon guidance. Science 274:1123–33 8895455 10.1126/science.274.5290.1123

[R118] Tigges J, Tigges M, Anschel S, Cross NA, Letbetter WD, McBride RL. 1981. Areal and laminar distribution of neurons interconnecting the central visual cortical areas 17, 18, 19, and MT in squirrel monkey (Saimiri). J. Comp. Neurol 202:539–60 7298914 10.1002/cne.902020407

[R119] Triplett JW, Phan A, Yamada J, Feldheim DA. 2012. Alignment of multimodal sensory input in the superior colliculus through a gradient-matching mechanism. J. Neurosci 32:5264–71 22496572 10.1523/JNEUROSCI.0240-12.2012 PMC3342701

[R120] Tzourio-Mazoyer N, De Schonen S, Crivello F, Reutter B, Aujard Y, Mazoyer B. 2002. Neural correlates of woman face processing by 2-month-old infants. NeuroImage 15:454–61 11798279 10.1006/nimg.2001.0979

[R121] van den Hurk J, Van Baelen M, Op de Beeck HP. 2017. Development of visual category selectivity in ventral visual cortex does not require visual experience. PNAS 114:E4501–10 28507127 10.1073/pnas.1612862114 PMC5465914

[R122] Vetter P, Bola L, Reich L, Bennett M, Muckli L, Amedi A. 2020. Decoding natural sounds in early “visual” cortex of congenitally blind individuals. Curr. Biol 30:3039–44.e2 32559449 10.1016/j.cub.2020.05.071 PMC7416107

[R123] Wallace MT, Perrault TJ Jr., Hairston WD, Stein BE. 2004. Visual experience is necessary for the development of multisensory integration. J. Neurosci 24:9580–84 15509745 10.1523/JNEUROSCI.2535-04.2004 PMC6730167

[R124] Wang L, Mruczek RE, Arcaro MJ, Kastner S. 2015. Probabilistic maps of visual topography in human cortex. Cereb. Cortex 25:3911–31 25452571 10.1093/cercor/bhu277 PMC4585523

[R125] Wolbers T, Klatzky RL, Loomis JM, Wutte MG, Giudice NA. 2011. Modality-independent coding of spatial layout in the human brain. Curr. Biol 21:984–89 21620708 10.1016/j.cub.2011.04.038 PMC3119034

[R126] Xu R, Bichot NP, Takahashi A, Desimone R. 2022. The cortical connectome of primate lateral prefrontal cortex. Neuron 110:312–27.e7 34739817 10.1016/j.neuron.2021.10.018 PMC8776613

[R127] Yeo BT, Krienen FM, Sepulcre J, Sabuncu MR, Lashkari D, 2011. The organization of the human cerebral cortex estimated by intrinsic functional connectivity. J. Neurophysiol 106:1125–65 21653723 10.1152/jn.00338.2011 PMC3174820

[R128] Yue X, Robert S, Ungerleider LG. 2020. Curvature processing in human visual cortical areas. NeuroImage 222:117295 10.1016/j.neuroimage.2020.117295 PMC7885662 32835823

[R129] Zeki S 1993. The visual association cortex. Curr. Opin. Neurobiol 3:155–59 8513225 10.1016/0959-4388(93)90203-b

